# GOBLET: The Global Organisation for Bioinformatics Learning, Education and Training

**DOI:** 10.1371/journal.pcbi.1004143

**Published:** 2015-04-09

**Authors:** Teresa K. Atwood, Erik Bongcam-Rudloff, Michelle E. Brazas, Manuel Corpas, Pascale Gaudet, Fran Lewitter, Nicola Mulder, Patricia M. Palagi, Maria Victoria Schneider, Celia W. G. van Gelder, GOBLET Consortium

**Affiliations:** 1 EMBnet, Nijmegen, The Netherlands; 2 SLU Global Bioinformatics Centre, Uppsala, Sweden; 3 Canadian Bioinformatics Workshops, Toronto, Canada; 4 ITICO, Cambridge, United Kingdom; 5 International Society for Biocuration, Geneva, Switzerland; 6 International Society for Computational Biology, San Diego, California, United States of America; 7 African Society for Bioinformatics & Computational Biology, Capetown, South Africa; 8 SIB Swiss Institute of Bioinformatics, Geneva, Switzerland; 9 The Genome Analysis Centre, Norwich, United Kingdom; 10 NBIC Netherlands Bioinformatics Centre, Nijmegen, The Netherlands; Ohio University, UNITED STATES

## Abstract

In recent years, high-throughput technologies have brought big data to the life sciences. The march of progress has been rapid, leaving in its wake a demand for courses in data analysis, data stewardship, computing fundamentals, etc., a need that universities have not yet been able to satisfy—paradoxically, many are actually closing “niche” bioinformatics courses at a time of critical need. The impact of this is being felt across continents, as many students and early-stage researchers are being left without appropriate skills to manage, analyse, and interpret their data with confidence. This situation has galvanised a group of scientists to address the problems on an international scale. For the first time, bioinformatics educators and trainers across the globe have come together to address common needs, rising above institutional and international boundaries to cooperate in sharing bioinformatics training expertise, experience, and resources, aiming to put ad hoc training practices on a more professional footing for the benefit of all.

## Introduction

Bioinformatics has become essential to the life sciences, especially important for supporting “omic” technologies. Now commonplace, these comprehensive studies bring new challenges (e.g., [1,2]), challenges that are likely to increase as genomic technologies enter the clinic and spawn even tougher data-generation-to-data-analysis issues. Already, the scale and complexity of the data is necessitating the use of sophisticated operating systems and database technologies, of command-line driven, custom-built algorithms that run on clusters or in high-performance or cloud-computing environments, and of rigorous statistical analysis tools. This has created a renewed demand for multi-disciplinary, “multi-lingual” individuals who have a deep understanding of the research domain, and who can talk to and work with computational scientists, statisticians, and engineers to tackle complex data stewardship and analysis tasks, and who can also acquire the necessary data storage, management, and analysis skills themselves.

Universities and research institutions are aware of this demand, but provision of bioinformatics training for life scientists is still patchy. Consequently, many students are not being equipped to get the most from currently available technologies. The problem stems partly from how much bioinformatics has changed, and partly from how it is taught. The latter can be especially difficult [3–5]: the field is broad, having grown from basic sequence and structure analysis to encompass a panoply of omic-related sub-disciplines; the audiences are diverse, ranging from those with biological backgrounds to those with foundations in mathematics and computer science; and the training needs vary, from those wanting to learn how to use particular tools or databases to those wanting to learn how to design, develop, and implement algorithms and software. Universities tend to focus on traditional disciplines, and can be reluctant to introduce new, cross-disciplinary courses, which often have small student cohorts relative to their mainstream counterparts. Some bioinformatics master’s programmes try to bridge the skills gap, but many of these also attract insufficient student numbers to remain viable. Hence, many bioinformatics courses fall outside, or have fallen off crowded university curricula, and much of the responsibility for providing bioinformatics training has fallen to other organisations, including national and international societies and networks, research institutes and research consortia.

Bioinformatics training needs are global, but training provision has been ad hoc, and repeatedly duplicated in a host of organisations across the world. Of course, having access to multiple training opportunities across the world is a good thing; however, no systematic endeavour had been made to coordinate these efforts. As a result, opportunities to share materials, courses, and even trainers, have been lost, and potential trainees have had no single place to find training information relevant to their needs. Each institution or country may have unique challenges, but there are many overlaps. It seemed likely, therefore, that global benefits could accrue for trainers, organisers, and trainees, were concerted efforts made to pool efforts and resources. With this harmonising vision and practical goals in mind, a group of like-minded scientists joined forces to create the Global Organisation for Bioinformatics Learning, Education and Training, (GOBLET, www.mygoblet.org). While “bioinformatics” is explicit in its name, the term is used in its widest sense to embrace aspects of biocuration, biocomputing, biostatistics, and computational biology—whatever is relevant to the maintenance, analysis, and interpretation of life-science data. In this paper, we present how and why GOBLET was established, its main achievements to date, and its future aspirations.

## Why GOBLET?

In 2009, the Bioinformatics Training Network (BTN) emerged as an outcome of SLING (Serving Life-science Information for the Next Generation), a European Seventh Framework Integrating Activity project. The BTN met regularly, discussing issues like the challenges and support requirements for bioinformatics training [6]; how to design and deliver a community-centred Web resource for sharing training materials [7]; and how to develop and share best working practices for creating short bioinformatics courses and for delivering bioinformatics training in general [8,9].

However, the bioinformatics training landscape clearly doesn’t stop at European borders. Virtually every bioinformatics and computational biology society, network, and institute includes some sort of education and training committee, each with a similar mission, and each with a similar problem: how to deliver tangible benefits with limited funds and a handful of volunteers? In 2012, representatives of ten such organisations met under the aegis of the 24th Annual General Meeting (AGM) of EMBnet (the Global Bioinformatics Network) to discuss this issue [10]. The rationale was that the diverse meeting participants would be uniquely placed to gain an overview of worldwide bioinformatics training activities and needs and to formulate a concerted strategy for addressing them.

The conclusion of the meeting was to create a single, dedicated foundation to allow the work of the BTN to continue beyond Europe. This was a vital decision, as funding for the BTN was coming to a close [11]. It was reasoned that a foundation like this would be resistant to the volatility of local institutional investment strategies and to the feast-or-famine nature of the funding afforded by typical grant cycles; it would therefore be better able to deliver on its aspirations for the benefit of its communities. A not-for-profit foundation—GOBLET—was therefore set up, and a kick-off meeting was held in November 2012, organised by an interim Executive Board and hosted by the Netherlands Bioinformatics Centre (NBIC) [12].

## GOBLET’s Ethos and Mission

Sharing knowledge and developing the bioinformatics skills of learners lie at the heart of GOBLET. This is reflected in the foundation’s ethos (which embraces inclusivity, sharing, and openness) and drives its mission to provide an open, sustainable support structure to foster the global community of bioinformatics trainers and trainees. The foundation has already amassed an international array of educators who are now sharing their expertise, best practices, and resources (adhering to Creative Commons Licences) across continents. They are also building synergistic relationships with users and beneficiaries, both via surveys and workshops, and through active dialogue with students and student bodies, for whom education and training are top priorities.

Providing bioinformatics education and training on a global scale is a challenge that will require time, focused effort, and innovative ideas. Members of GOBLET are embracing this challenge in a collective effort to make bioinformatics knowledge and skills available for all. Many training ventures will, of course, require local solutions that use local resources, and part of the challenge will be to help design training materials and programmes for countries with varying levels of infrastructure support. Nevertheless, with its global perspective and the combined experience and expertise of its members, GOBLET is ideally and uniquely placed to discuss, devise, and implement appropriate solutions.

## Who GOBLET Is For

A variety of stakeholders stand to benefit from the cooperative, collaborative framework that GOBLET provides, albeit in different ways:

### Organisations

As an organisation of organisations, GOBLET largely comprises organisational members (societies, networks, institutes, etc.). For them, benefits include opportunities to network with trainers from other organisations; to identify other trainers with whom to work; to publicise training events; and to acquire coordinated views of the training landscape. By exploiting GOBLET, organisations may share existing materials, curricula, etc., and avoid duplicating the same resources in-house. As part of a consortium with a critical mass of respected national and international members, organisations may also cooperate in developing coordinated funding proposals and work together to help shape future support policies for bioinformatics training.

### Research Groups and Consortia

The same is true for smaller research groups and consortia; for them, however, particular advantages are the gearing that may be achieved from interaction with a much larger, established community of trainers and the wider opportunities this may afford to participate in global training events and to discover potential future collaborators.

### Corporations

In addition to these benefits, what may be particularly beneficial to companies is the ability to use GOBLET as a platform to express the specific training needs of industry, and to increase the visibility of the industrial sector, either as training consumers or as training providers; for them, GOBLET may also provide an opportunity for establishing training collaborations.

### Funders

Increasingly, delivery of bioinformatics training is being embedded in grant awards in the life sciences. For funding bodies, then, the foundation offers a partner organisation with which to discuss, coordinate and identify relevant training needs and resources, and to help formulate policies on how best to support bioinformatics training in the future. In turn, GOBLET offers a global platform for advertising training activities that such bodies support.

### Organisers and Trainers

GOBLET grew from a vision of organisers and trainers to harmonise bioinformatics training globally. Trainers may now profit from existing experience on course design, from recommendations of trainers, IT infrastructures, etc.; they may exchange training materials and methods, and gain inspiration from those of others; they may learn about pedagogical advances, and discuss the pros and cons of different training approaches. Similarly, organisers may promote their courses, share and disseminate their resources via a single portal, devise new courses to plug training gaps, discuss the challenges they face, and involve trainers in mitigating some of the issues.

### Trainees

The main value for trainees is access to an organised, professional network of trainers and their know-how. Those wanting to take specific courses benefit from being able to consult a single portal with the latest news on local and globally distributed training events, where they may download relevant tutorials, case studies, data-sets, etc. By becoming involved, trainees may use GOBLET to express their training requirements, to influence future development of bioinformatics training materials, and to lobby for core bioinformatics topics that they believe should make it onto course curricula and degree programmes.

## GOBLET’s Priorities

The foundation’s priorities fall into several broad themes: these encompass *quality* of training, of training resources, etc.; training *accreditation* and *recognition* for training provision; methodology in training best practices; training *outreach;* and financial *sustainability* of global bioinformatics education initiatives. The short-term focus is to evolve the training portal into a global, community-centred resource and to support activities to help develop the next generation of bioinformaticians, including providing training in geographically remote and infrastructure-poor areas through trainer exchanges. GOBLET is thus gathering knowledge from users to inform and guide its future directions, working collaboratively to ensure synergies with related training activities, and actively scouting for key stakeholders and funding opportunities.

Longer-term aims include plugging gaps where there is known to be a dearth of training materials (e.g., in standards development and use of standards in biocuration [1], or in regions where training opportunities are limited (e.g., [13,14]). Ultimately, GOBLET aims to develop and maintain high-quality, comprehensive, branded products (lecture materials, exercises, data-sets, etc.) and to begin to implement accreditation mechanisms. For the future, GOBLET is also discussing the creation of a harmonised bioinformatics and computational biology education conference with the International Society for Computational Biology (ISCB).

## Governance Structure and Members

**Fig 1:**
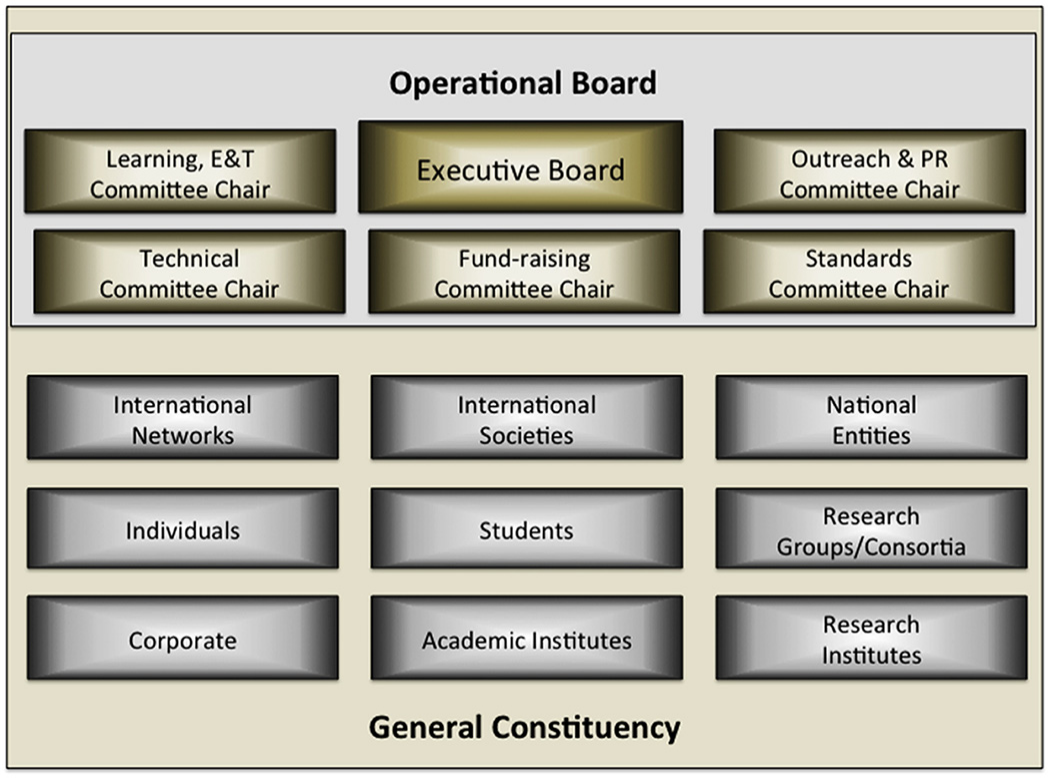
Governance structure. The operational board comprises the executive board and committee chairs. The general constituency includes representatives of member organisations, and individuals.

These priority areas suggested a natural framework for organising GOBLET’s work, and five committees were duly established as part of a formal governance structure (Fig 1). The largest, the Learning, Education and Training Committee (LET), is responsible for developing and sharing best practices in teaching and learning methods. This committee is also examining curriculum guidance, exploring trainer recognition mechanisms and investigating ways of providing support structures for trainers. Working with the Standards Committee, LET is investigating accreditation mechanisms and considering ways generally to drive up the quality of training materials and courses delivered and deposited in the repository. Both committees work with the Technical Committee to shape GOBLET’s overarching support infrastructure for course organisers, trainers, and trainees.

To try to sustain the foundation’s work, the Fund-raising Committee is tasked with stimulating collaborative projects within GOBLET and with other training communities. Alongside this work, the Outreach and PR (public relations) Committee is responsible for promoting GOBLET, maintaining its social networking interactions (twitter @mygobletorg) and galvanising communities to participate in its initiatives.

Overall, the foundation’s work is orchestrated by an executive board, which liaises with the committee chairs to form an operational board that ensures that the foundation’s activities are in harmony with the foundation’s core mission, aligned with the needs of trainees worldwide and, importantly, in line with its own statutes and bylaws. These Boards report formally to the full constituency at AGMs. Committee chairs and members of the first formal executive board were elected in autumn 2013, and took up their roles at the first AGM in November 2013 [15].

Supported by this governance structure, GOBLET’s work is driven by its members. The membership categories, mentioned earlier (Fig 1), include international societies and networks, national entities, corporate bodies, and individuals (including students). All members may join the foundation’s activities via its committees; organisational members may also hold executive office, and hence be instrumental in steering GOBLET’s current and future development.

## Achievements to Date

A wave of enthusiasm arose with the creation of GOBLET, a testament to the keenly felt need to galvanise national and international bioinformatics training communities. Since its inception, there have been several notable achievements:

### Engaging with and Building Communities

To engage with likely beneficiaries, we began by working with the Society for Experimental Biology (SEB) to solicit the type and level of bioinformatics training that biologists need to facilitate their work. The results were of sufficient interest to push the survey out more widely through GOBLET partner organisations, to see whether the initial findings were general across global communities. The outcomes were presented at a satellite workshop at the SEB 2014 Annual Meeting, where reactions to the results were gathered, additional issues identified, and ideas for prioritising and addressing the training needs and gaps were discussed [16]. This feedback was discussed at the 2014 AGM, and is currently being collated in a “state of the field” paper.

We also worked with the ISCB to create an education poster track for the Intelligent Systems for Molecular Biology (ISMB) international conferences (GOBLET had submissions accepted in 2013 and 2014). A particular highlight at ISMB 2014 was the launch of a Community of Special Interest (COSI) around Computational Biology Education (CoBE), aiming to provide a supportive environment where ISCB and GOBLET communities can work together and reach out to those with similar interests (cosi.iscb.org/wiki/CoBE:Home). Led by the ISCB Education committee, another collaborative activity here was a teacher-training workshop to help bring bioinformatics activities into high school classrooms, an activity that has been growing and where there is increasing interest among teachers [17].

Many other community-building events and workshops have been held (Table 1). These have allowed us to discuss themes such as design of bioinformatics and computational biology curricula, and the practicalities of launching global student intern initiatives and multi-disciplinary, multi-institutional PhD programmes; they have also allowed the GOBLET and ELIXIR communities to meet, to share training experiences and to discuss the scalable actions needed to implement a pan-European bioinformatics training strategy (ELIXIR is a distributed, European research infrastructure for life-science information, an inter-governmental initiative seeded by the European Strategy Forum on Research Infrastructures. Alongside its goal to collect and archive large amounts of life-science data, ELIXIR aims to upskill researchers by training them to exploit its data, tools, standards and compute services more effectively). In addition, GOBLET members have acted as ambassadors in diverse meetings and workshops around the world, including venues in Europe, Africa, the United States, and Australia.

**Table 1:** GOBLET meetings and workshops.

Date	Host/Location	Description
June 2012	EMBnet AGM, Uppsala, SE	Inaugural meeting to discuss the formation of a bioinformatics training foundation
November 2012	NBIC, Amsterdam, NL	GOBLET 2012, 1st formal meeting
March 2013	The Genome Analysis Centre (TGAC), Norwich, UK	ELIXIR-UK/GOBLET Workshop
July 2013	ISCB, Berlin, DE	Workshop on bioinformatics education
November 2013	TGAC, Norwich, UK	Pan-European bioinformatics training strategy workshop
November 2013	TGAC, Norwich, UK	GOBLET 2013 AGM
July 2014	SEB Annual Meeting, Manchester, UK	Bioinformatics workshop—an essential tool for experimental biologists
July 2014	ISMB 2014, Boston, US	High-school teacher workshop
November 2014	Bioinformatics.ca, Toronto, CA	GOBLET 2014 AGM

### Training Portal

Early on, we launched a training portal, which we are beginning to shape into a significant community asset, providing a non-redundant, centralised repository of bioinformatics training information, courses, materials, etc., accessible to all [18]. We have worked with organisations like the SEB to solicit user feedback and ensure that this addresses real user needs, and are liaising with ELIXIR to avoid duplication of effort and harmonise their nascent training portal with the resource currently being delivered by GOBLET.

### Fundraising

For international groups, face-to-face meetings are expensive. We were thus fortunate to have Bioinformatics.ca win a grant from the Canadian Institutes for Health Research to help support GOBLET’s 2014 AGM in Toronto. In the very positive feedback received, one reviewer commented, “This proposal seeks… to develop an Action Plan to coordinate global bioinformatics training. This is an important undertaking…and a critical step forward.” Another success was a small Australian Bioinformatics Network Connection Grant to fund a GOBLET speaker to headline an education session at the 13th International Conference on Bioinformatics (InCoB 2014) in Sydney. Such efforts bear witness to the importance attached to the international dimension that GOBLET provides.

## Getting Involved

GOBLET offers an opportunity to become part of an active, worldwide community of bioinformatics educators and trainers. Since the 10 founding partners met in 2012, membership has increased 4-fold, including 28 national and international organisations. Further success depends on global participation, so we warmly encourage all relevant institutions and motivated individuals across continents to join the foundation and its activities—the wider the membership, the broader our perspective will be on global challenges in bioinformatics education and training, and the greater our ability to address them. But perhaps amongst the greatest rewards and incentives are the inspiration that comes from working towards common goals, the *leveraging* such cooperation brings (when pulling together policy or standards documents, writing opinion papers and grant applications, etc.), and the certain knowledge that access to better coordinated, better quality training must, in due course, enable better research—in this context, GOBLET’s whole is undoubtedly greater than the sum of its parts.

GOBLET was created as an umbrella organisation, to harmonise worldwide bioinformatics training activities, and to allow its members to work together more powerfully towards a sustainable future. To this end, the foundation exploits a mixed funding model (including grants, donations, and member subscriptions), in order to withstand funding deserts or sudden changes in institutional funding policies. To try to be inclusive, the membership structure offers various tiers, each with specific benefits (details at www.mygoblet.org). This structure affords significant flexibility, and can cater to both individuals and organisations of different sizes, from small research groups to large international organisations and corporations. The 2015 GOBLET AGM will be held in Cape Town, 18–20 November (full details to be published on the website, as they emerge). Alongside members, we also encourage observers to attend what will undoubtedly be a stimulating meeting.

To date, GOBLET has been able to subsidise meetings, to fund outreach and PR activities (including speakers and posters at meetings and conferences), and to maintain the training portal. As it matures, the foundation aims to support an increasing portfolio of activities: e.g., publication of articles, guidelines and opinion pieces; production and maintenance of high-quality, comprehensive training materials and data-sets; an annual conference; further outreach to schools; and perhaps also a discussion forum, especially to engage with and benefit students/early-stage researchers. We welcome wider input to these exciting initiatives, to collectively nurture the GOBLET foundation, and ultimately help it to evolve into the professional body for bioinformatics educators and trainers it aspires to be.

## References

[1] Howe D, Costanzo M, Fey P, Gojobori T, Hannick L, et al (2008) Big data: The future of biocuration. Nature 455: 47–50. 10.1038/455047a 18769432 PMC2819144

[2] Ouzounis CA (2012) Rise and demise of bioinformatics? Promise and progress. PLoS Comput Biol 8: e1002487 10.1371/journal.pcbi.1002487 22570600 PMC3343106

[3] Pevzner PA (2004) Educating biologists in the 21st century: bioinformatics scientists versus bioinformatics technicians. Bioinformatics 20: 2159–2161. 15073013 10.1093/bioinformatics/bth217

[4] Hack C, Kendall G (2005) Bioinformatics: Current practice and future challenges for life science education. Biochem Mol Biol Educ 33: 82–85. 10.1002/bmb.2005.494033022424 21638550

[5] Zeti AMH, Shamsir MS, Tajul-Arifin K, Merican AF, Mohamed R, et al (2009) Bioinformatics in Malaysia: hope, initiative, effort, reality, and challenges. PLoS Comput Biol 5: e1000457 10.1371/journal.pcbi.1000457 19714208 PMC2723929

[6] Schneider MV, Watson J, Attwood T, Rother K, Budd A, et al (2010) Bioinformatics training: a review of challenges, actions and support requirements. Brief Bioinform 11: 544–551. 10.1093/bib/bbq021 20562256

[7] Schneider M V, Walter P, Blatter M-C, Watson J, Brazas MD, et al (2012) Bioinformatics Training Network (BTN): a community resource for bioinformatics trainers. Brief Bioinform 13: 383–389. 10.1093/bib/bbr064 22110242 PMC3357490

[8] Via A, De Las Rivas J, Attwood TK, Landsman D, Brazas MD, et al (2011) Ten simple rules for developing a short bioinformatics training course. PLoS Comput Biol 7: e1002245 10.1371/journal.pcbi.1002245 22046119 PMC3203054

[9] Via A, Blicher T, Bongcam-Rudloff E, Brazas MD, Brooksbank C, et al (2013) Best practices in bioinformatics training for life scientists. Brief Bioinform 14: 528–537. 10.1093/bib/bbt043 23803301 PMC3771230

[10] GOBLET Consortium. (2013) The Global Organisation for Bioinformatics Learning, Education and Training (GOBLET). EMBnet.journal 19(1), 10–13.

[11] Meeting Report—June (2012) http://www.mygoblet.org/sites/default/files/goblet_events/B3CBFollowUp-DraftReport2.pdf

[12] Meeting Report – November (2012) http://www.mygoblet.org/sites/default/files/GOBLETminutes281112_version191212-TA.pdf

[13] Orozco A, Morera J, Jiménez S, Boza R (2013) A review of bioinformatics training applied to research in molecular medicine, agriculture and biodiversity in Costa Rica and Central America. Brief Bioinform 14: 661–670. 10.1093/bib/bbt033 23723382

[14] Tastan Bishop O, Adebiyi EF, Alzohairy AM, Everett D, Ghedira K, et al (2014) Bioinformatics Education-Perspectives and Challenges out of Africa. Brief Bioinform. 10.1093/bib/bbu022 PMC4364068 24990350

[15] Meeting Report – November (2013) http://www.mygoblet.org/sites/default/files/goblet_events/GOBLET-AGMReportExecSummary.pdf

[16] Meeting Report – July, (2014) http://www.mygoblet.org/sites/default/files/goblet_events/SEBGOBLETWorkshopReport070714.pdf

[17] Machluf Y, Yarden A (2013) Integrating bioinformatics into senior high school: design principles and implications. Brief Bioinform 14: 648–660. 10.1093/bib/bbt030 23665511

[18] Corpas M, Jimenez RC, Bongcam-Rudloff E, Budd A, Brazas MD, et al (2014) The GOBLET training portal: a global repository of bioinformatics training materials, courses and trainers. Bioinformatics 31: 140–142. 10.1093/bioinformatics/btu601 25189782 PMC4271145

