# Correction: GOBLET: The Global Organisation for Bioinformatics Learning, Education and Training

**DOI:** 10.1371/journal.pcbi.1004281

**Published:** 2015-05-14

**Authors:** Teresa K. Attwood, Erik Bongcam-Rudloff, Michelle E. Brazas, Manuel Corpas, Pascale Gaudet, Fran Lewitter, Nicola Mulder, Patricia M. Palagi, Maria Victoria Schneider, Celia W. G. van Gelder, GOBLET Consortium

The first author’s surname is spelled incorrectly. The correct name is Teresa K. Attwood.
